# Bioactive Substances of Potato Juice Reveal Synergy in Cytotoxic Activity against Cancer Cells of Digestive System Studied *In Vitro*

**DOI:** 10.3390/nu15010114

**Published:** 2022-12-26

**Authors:** Przemysław Łukasz Kowalczewski, Anna Olejnik, Martyna Natalia Wieczorek, Joanna Zembrzuska, Katarzyna Kowalska, Jacek Lewandowicz, Grażyna Lewandowicz

**Affiliations:** 1 Department of Food Technology of Plant Origin, Poznań University of Life Sciences, 31 Wojska Polskiego St., 60-624 Poznań, Poland; 2 Department of Biotechnology and Food Microbiology, Poznań University of Life Sciences, 48 Wojska Polskiego St., 60-627 Poznań, Poland; 3 Institute of Chemistry and Technical Electrochemistry, Poznan University of Technology, 4 Berdychowo St., 60-965 Poznań, Poland; 4 Institute of Logistics, Poznan University of Technology, 2 Jacka Rychlewskiego St., 60-965 Poznań, Poland

**Keywords:** solanine, chaconine, phenolic acids, biological activity, *Solanum tuberosum*

## Abstract

More and more literature data indicate the health-promoting effect of potato juice (PJ). However, to date, it has not been precisely explained which of the many compounds present in PJ exhibit biological activity. The work aimed to establish the antiproliferative effect of gastrointestinal digested PJ and the products of its processing. Fresh PJs derived from three edible potato varieties, industrial side stream resulting from starch production, partially deproteinized PJ derived from feed protein production line, and three different potato protein preparations subjected to digestion in the artificial gastrointestinal tract were used in this study. The cytotoxic potential of glycoalkaloids (GAs), phenolic acids, digested PJ, and products of PJ processing was determined in human normal and cancer cells derived from the digestive system. The results showed that GAs exhibit concentration-dependent cytotoxicity against all analyzed cell lines. In contrast, phenolic acids (caffeic, ferulic, and chlorogenic acid) do not show cytotoxicity in the applied cell lines. A correlation between cytotoxic potency and GAs content was found in all PJ products studied. The most potent effects were observed under treatment with deproteinized PJ, a product of industrial processing of PJ, distinguished by the highest effective activity among the fresh juice products studied. Moreover, this preparation revealed a favorable cytotoxicity ratio towards cancer cells compared to normal cells. Statistical analysis of the obtained results showed the synergistic effect of other bioactive substances contained in PJ and its products, which may be crucial in further research on the possibility of using PJ as a source of compounds of therapeutic importance.

## 1. Introduction

Potato juice (PJ) can be simply defined as a liquid fraction of potato tubers. It is a complex mixture that contains approximately 6% of dry mass. The main fractions of potato juice are proteinaceous compounds (amino acids, peptides, and proteins) of high nutritional value. Apart from them, PJ also contains other organic compounds, such as sugars, carboxylic acids, or vitamins, as well as approximately 1% of minerals. On an industrial scale, PJ is generated by the production of starch. This side stream was initially considered a troublesome waste and the driving force behind the development of the technology of its valorization for environmental sakes [1,2]. Currently, due to the huge increase in interest in new, vegetable sources of protein, there has been significant technological progress in processing PJ. Moreover, starch-producing companies consider the production of potato protein for human nutrition a task as important as the production of starch itself [3,4,5,6,7]. However, the value of PJ is not limited to human nutrition. Current literature data indicate various biological activities, including antioxidant, antimicrobial, anti-inflammatory, anticancer, antiobesity, antidiabetic, antihyperlipidemic, and antihypertensive effects [7,8,9,10,11,12,13,14,15,16]. The high medicinal potential of PJ is also supported by the long-standing tradition in European folk medicine of using this substance for the treatment of gastric ulcers [17,18]. As the diagnosis and differentiation of diseases in folk medicine were extremely limited, it is currently difficult to refer to what types of diseases were treated with potato juice. It was probably a series of diseases of various etiology, including neoplastic changes.

PJ contains a lot of substances with biological activity. These are both proteins or peptides (mainly protease inhibitors), as well as a lot of various non-proteinaceous substances. Among the latter, vitamin C and the vitamin B complex should be mentioned, as well as polyphenols, mainly: chlorogenic and caffeic acids, lutein, violaxanthin, zeaxanthin, antheraxanthin, and β-carotene [19,20]. Special attention should be paid to glycoalkaloids (GAs), mainly α-chaconine and α-solanine, which are commonly known for their harmful effects on the human body. The maximum level of glycoalkaloids in the potato protein preparations introduced in the European market has been set at 150 mg per 1 kg [21] to protect consumers’ health against poisoning [22]. Nevertheless, literature data suggests a huge medicinal potential of gas, especially in cancer treatment [23]. *In vitro* studies have demonstrated the antiproliferative effect of α-solanine against different human tumor cell lines: HepG2 (liver), AGS and KATO III (stomach), HT-29 (colon), Panc-1 and SW1990 (pancreas), U937 (lymphoma), Jurkat (leukemia), A2058 (melanoma), PC-3 and DU145 (prostate), RL95-2 (endometrium), EC9706 (esophagus) and HeLa (cervix) [23,24,25]. What is important, it was shown that although α-solanine can be toxic to normal cells at higher doses, it may reveal therapeutic effects against cancer cells at non-toxic concentrations [26]. These observations were also verified in in vivo studies [27,28]. Similar results were also obtained in *in vitro* studies with α-chaconine using colon (HT-29), liver (HepG2), cervix (HeLa), lymphoma (U937), and stomach cell lines [24,25,29]. What is important is that the simultaneous use of both GAs may cause a synergistic effect [29]. *In vitro* studies have also shown the antitumor activity of other components of potato juice, including polyphenols (mainly chlorogenic acid) and protease inhibitors (one of the juice’s protein fractions) [30,31,32].

This above-presented short description of the current knowledge regarding the medicinal potential of potato juice points to the possibility of using PJ or its components for cancer treatment or at least to support the treatment by diet fortification. Attempts to use potato juice for the production of functional foods have already been undertaken, and their therapeutic use has been demonstrated in short, preliminary clinical trials [33,34,35,36]. A barrier to the wider use of PJ and its processing products in supporting cancer treatment is the lack of knowledge of the interactions of individual PJ components. In particular, it is not known whether the individual components of the PJ show a synergistic effect or, on the contrary, they have an antagonistic effect. The changes that PJ undergoes during digestion and how this affects its biological activity, as well as the effect of the processing of PJ on its bioactivity, are also not known.

Given the aforementioned, the foundation of our study was to verify whether the bioactive compounds present in PJ show mutual interaction in the context of cytotoxicity. Thus, the work aimed to establish the antiproliferative effect of gastrointestinal digested PJ and the products of its processing. Fresh PJs derived from three different potato varieties, industrial side stream resulted from starch production, partially deproteinated PJ derived from feed protein production line, and three different potato protein preparations subjected to digestion in the artificial gastrointestinal tract were used in this study.

## 2. Materials and Methods

### 2.1. Experimental Materials

Fresh juices from potatoes of 3 varieties (‘Agata’, ‘Queen Anne’ and ‘Vivaldi’, denoted as ‘APJ’, ‘QAPJ’ and ‘VPJ’, respectively) were obtained by thoroughly washing and peeling the tubers, and then squeezing the juice using a VitaJuice 4 juicer (Robert Bosch GmbH, Gerlingen-Schillerhöhe, Germany). Each time, the juice obtained was centrifuged at 4 °C (3000× *g*) in order to separate the starch remaining in the juice, and then the juice was decanted, frozen, and freeze-dried.

Industrial potato juice (IPJ) and deproteinized potato juice water (DPJ) were collected from PPZ Trzemeszno S.A. (Trzemeszno, Poland) as a side stream of the potato starch extraction process.

Moreover, the research used potato juice protein concentrate (MPP), obtained according to the membrane method described in detail earlier [6], commercial potato protein Solanic^®^200 (NPP) purchased from Avebe (Veendam, The Netherlands), and feed potato protein (FPP) obtained from PPZ Trzemeszno S.A. (Trzemeszno, Poland).

α-Solanine and α-chaconine were purchased from Phytolab GmbH & Co. KG (Vestenbergsgreuth, Germany). Pork pepsin, porcine pancreatin, bile salt, caffeic, chlorogenic, ferulic, and gallic acids were purchased from Sigma-Aldrich (Steinheim, Germany). All other reagents, purity min. HPLC grades were purchased from Merck Life Science (Darmstadt, Germany).

### 2.2. Digestion Process

The *in vitro* gastrointestinal digestion procedure was performed with a simplified methodology on the basis of the method previously described in detail by Olejnik et al. [37]. In short, the digestion process of analyzed samples was performed in a glass bioreactor, which was thermally stable, and the reactions were carried out at 37 °C. Samples for further analyses were prepared by taking 10 g of freeze-dried experimental products and dissolving them in demineralized water to a volume of 100 mL. Independent products of digestion were obtained after digestion in the stomach (denoted as a suffix -G) and also after complete gastrointestinal digestion (denoted as -GI). Stomach stage: with 1 M HCl, the pH of the digested mixture was lowered to 2.0, and then a solution of pork pepsin in 0.1 M HCl was added to obtain a concentration of 1.92 mg pepsin/mL of the mixture. The process was carried out for 2 h with constant stirring. Intestine stage: After a 2-h gastric digestion, the pH of the mixture was raised to 7.0 with 2M NaHCO_3_ and then supplemented with porcine pancreatin (0.4 mg/mL) and bile salt (2.4 mg/mL). Intestinal digestion was carried out for 2.5 h. All digestion samples were frozen at −80 °C and then lyophilized. In order to perform the quantitative analysis, the volumes of the digested mixtures were monitored at all stages of the gastrointestinal tract. The digestion process described above was performed in triplicate using the same experimental material.

### 2.3. GAs Content

The isolation and purification of the GAs from the analyzed samples were carried out according to the procedure described in detail previously [38]. In short, lyophilized samples were extracted with 5% acetic acid, shaken for 15 min, and then centrifuged (10,000× *g*, 15 min, 4 °C). The GAs were isolated from the supernatant obtained using solid phase extraction (SPE) cartridges (HLB Oasis 1cc 30 mg, Waters Corporation, Milford, MA, USA), eluting with methanol with formic acid (0.1% *v/v*). The eluate was filtered (0.22 µm) before the analysis.

The quantitative and qualitative determination of *α*-solanine and *α*-chaconine was performed using the chromatographic system UltiMate 3000 RSLC (Dionex™, Thermo Scientific Inc., Waltham, MA, USA) coupled to an API 4000 QTRAP triple quadrupole mass spectrometer with electrospray ionization (ESI) (from AB Sciex, Foster City, CA, USA) in positive ionization mode (UHPLC–MS/MS). Chromatographic separation was performed on a Kinetex 1.7 µm C18 column (100 mm × 2.1 mm I.D.) from Phenomenex Inc., Torrance, CA, USA. 0.1% formic acid (A) and acetonitrile (B) were used as the mobile phase. Elution was performed using a gradient: 25% B at 0 min, 32% at 3 min, increased to 100% B in 3 min, and held for 0.5 min. The flow rate was 0.20 mL/min. The column temperature was maintained at 35 °C, and the injection volume was 10.0 μL. A post-run time was set at 4.0 min for column equilibration before the next injection. The operating conditions for mass spectrometry for *α*-solanine and *α*-chaconine were as follows: curtain gas 10 psi, nebulizer gas, and auxiliary gas 40 psi, source temperature 600 °C, ion spray voltage 5500 V, and collision gas set to medium. Quantitative analysis of the compounds was performed in multiple reaction monitoring (MRM) mode, for analytes were chosen one transition of the protonated molecular ion and their respective ion product. The first MRM transition was used to quantitate; the second was used as confirmation. These transitions (*m/z*) with associated decluttering potentials (V) and collision energies (V) were: *α*-solanine 869 → 98, 181, 115; 869 → 398, 181, 95 and *α*-chaconine 852.6 → 98, 201, 119; 852.6 →706, 201, 97.

### 2.4. Polyphenols Profile

Extraction of the polyphenolic compounds was done using 80% methanol. The methanol solution was added to 150 mg of the lyophilized samples to obtain a volume of 1.5 mL. The samples prepared in this way were shaken for 20 min, centrifuged (10,000× *g*, 10 min, 4 °C), and the obtained supernatants were filtered through a 0.22 µm filter.

The same LC-MS/MS system, as for *α*-solanine and *α*-chaconine determination, was used for the determination of phenolic acids (PAs), including caffeic (CaA), chlorogenic (ChA), and ferulic (FA) acids, according to the method described by Cybulska et al. [39]. Chromatographic separation was achieved on the analytical column Luna 3 µm C18 (150 mm × 2.0 mm I.D., Phenomenex Inc., Torrance, CA, USA) using 5 mM ammonium acetate in water (A) and methanol (B) as mobile phase in a gradient mode of elution: 0 min 50% B, 2.5 min 50% B, 3 min 100% B, and 3.5 min 100% B. The flow rate was 0.2 mL/min, and the injection volume was 5.0 μL. MS-MS detection was performed in a negative ionization mode with the ion transitions (*m/z*) with associated decluttering potential (V) and collision energies (V) were: for caffeic acid 179 → 135, −51, −22; 179 → 106, −51, −32, for chlorogenic acid: 353 → 85, −65, −24; 353 → 191, −65, −64, for ferulic acid: 193 → 134, −55, −20; 193 → 178, −55, −18, and for gallic acid: 169 → 125, −55, −22; 169 → 79, −32, −5. The analyte was detected using the following settings for the ion source and mass spectrometer: curtain gas 10 psi, nebulizer gas 40 psi, auxiliary gas 40 psi, temperature 400 °C, ion spray voltage −4500 V, and collision gas set to medium. All compounds were quantified in extracts using the standard addition method.

### 2.5. In Vitro Study

#### 2.5.1. Cell Cultures

The cytotoxic potential of potato GAs (α-chaconine and α-solanine), PAs (CA, ChA, FA), and digested PJ and products of its processing (PDPJPs) were determined in cancer and normal human cells derived from the digestive system. The normal small intestine hIEC-6 (ATCC^®^ CRL-3266™) and colon mucosa CCD 841 CoN (ATCC^®^ CRL-179™) cell lines, gastric carcinoma AGS (ATCC^®^ CRL-1739™) and Hs 746T (ATCC^®^ HTB-135™) cell lines, colorectal adenocarcinoma Caco-2 (ATCC^®^ HTB-37™) and HT-29 (ATCC^®^ HTB-38™) cell lines were obtained from the American Type Culture Collection (ATCC, Manassas, VA, USA). Cell lines were cultured under standard conditions recommended as optimal by ATCC.

#### 2.5.2. Cytotoxicity Assay

In the cytotoxicity tests, the cells were grown in 96-well plates at an initial density of 1.5 × 10^4^ cells/cm^2^. The 24-h cultures were treated with GAs (1–20 µM), PAs (10–200 µM), and PDPJPs (0.1–20 mg/mL) for 48 h. The range of GAs doses was established considering GAs solubility and cytotoxicity to all cell cultures tested. GAs dose range included non-toxic and highly toxic (lethal) GAs concentrations in human normal and cancer cell lines. In the cytotoxicity analysis of potato phenolic acids, concentrations up to 200 μM were applied due to their physiological relevance and contents in PJ products. DPJP concentration range was established to allow modeling of the cell response-dose curve and calculation of half maximum cytotoxic doses. The exposition time, which should not be less than the time needed to double the cell population, was optimized for all cell lines used in the experiments. As the proliferation rate of the cells derived from normal tissues was significantly lower than that of cells isolated from tumor tissues and the doubling population time determined in normal intestinal cell cultures exceeded 24 h, the treatment time was established at 48 h. The effect of the potato compounds and products on cell viability and metabolic activity was evaluated with the MTT (3-(4,5-dimethylthiazol-2-yl)-2,5-diphenyltetrazolium bromide (Sigma–Aldrich, Steinheim, Germany) test according to the previously described procedure [40]. This test was chosen because of its high sensitivity and repeatability. Preliminary experiments showed that the MTT test was more sensitive and accurate than alternative cytotoxicity assays, including the Sulforhodamine B and Alamarblue™ tests. After normal and cancer cell treatment, the MTT solution (5 mg MTT/mL) was added to each well to obtain a concentration of 0.5 mg MTT/mL. The cultures were incubated at standard culture conditions for 3 h, and then formazan crystals were extracted with isopropanol for 20 min at room temperature. The absorbance was measured at 570 nm and 690 nm using a Tecan M200 Infinite microplate reader (Tecan Group Ltd., Männedorf, Switzerland). Based on the MTT results, dose-response curves were plotted, and then cytotoxic doses of the analyzed compounds were calculated.

### 2.6. Statistical Analysis

The experimental data, presented as mean ± SD, was studied using a one-way analysis of variance, and the Tukey post hoc test was used to determine statistically homogenous subsets at α = 0.05. The Pearson correlation coefficient was calculated for GAs content in products before and after gastric and gastrointestinal digestion. Principal component analysis was performed based on a correlation matrix. Clustering was performed based on Ward’s method, and Euclidean distance was used as a measure of similarity. Statistical analyses were performed using Statistica 13.3 software (Dell Software Inc., Round Rock, TX, USA).

## 3. Results and Discussion

### 3.1. Cytotoxicity of Individual Bioactive Compounds

**Figure 1:**
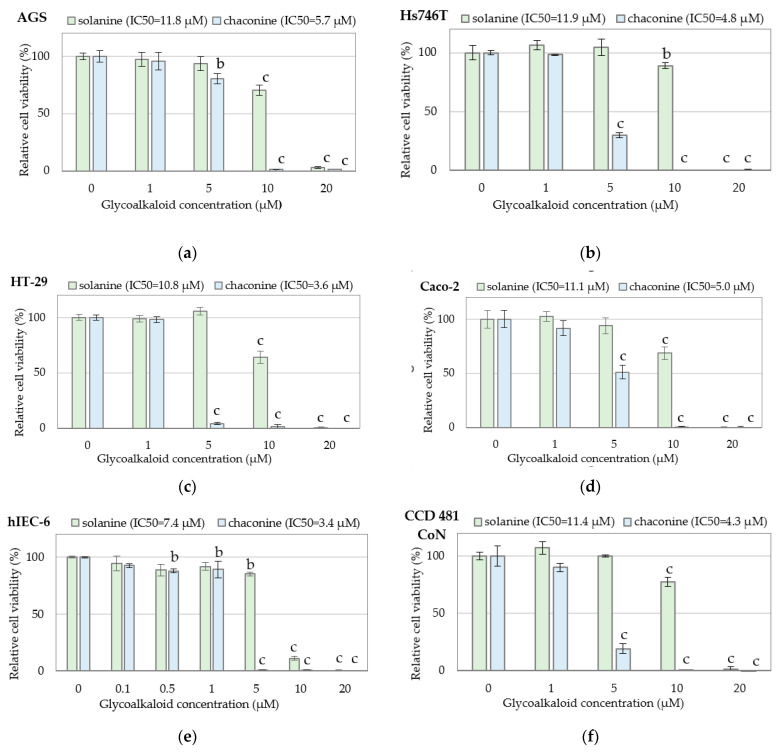
Cytotoxicity of glycoalkaloids against cancer and normal cells: (**a**) gastric cancer AGS cells; (**b**) gastric cancer Hs746T cells; (**c**) colon cancer HT-29 cells; (**d**) colon cancer Caco-2 cells; (**e**) normal small intestine hIEC-6 cells; (**f**) normal colon mucosa CCD 481 CoN cells determined by the MTT test. Changes in cell viability under GA treatment were expressed relative to control cell culture non-treated with GA tested. Values represent the means ± SD (n = 3). ‘b’ *p* < 0.01, ‘c’ *p* < 0.001 (Tukey’s post hoc test).

As far as the cytotoxic activity of individual bioactive compounds in PJ is concerned, most studies have been published regarding toxic GAs and PAs that are considered health-promoting [7,23]. With regard to the latter, particular attention has been paid to chlorogenic acid [30]. Our experiment proved that GAs exhibit concentration-dependent cytotoxicity against gastric and colon cancer cells. The results of GAs cytotoxicity were presented in Figure 1, including cytotoxicity to gastric cancer AGS (Figure 1a) and Hs746T (Figure 1b), to colon cancer HT-29 (Figure 1c) and Caco-2 (Figure 1d) cells, and normal small intestine hIEC-6 cells (Figure 1e) and normal colon mucosa CCD 481 CoN cells (Figure 1f). Chaconine turned out to be more toxic. Chaconine at a concentration of 5 µM evoked strong cytotoxic effects, whereas solanine at this concentration did not significantly decrease the viability of the analyzed cells. The distinctly different cytotoxic effects of solanine and chaconine at a dose of 5 µM are especially evident in the HT-29 cell line (Figure 1c). Similar observations were made in normal non-cancerous cells of the small intestine and colon (Figure 1e,f). The obtained results are consistent with the literature data, which also indicates higher toxicity of chaconine than solanine [41,42].

Based on the obtained data, the values of the half maximal inhibitory concentration (IC_50_) of GAs were calculated and presented in Table 1. Solanine IC_50_ doses confirm its lower cytotoxic potential than chaconine. Normal intestinal hIEC-6 cells were most sensitive to both GAs.

**Figure 2:**
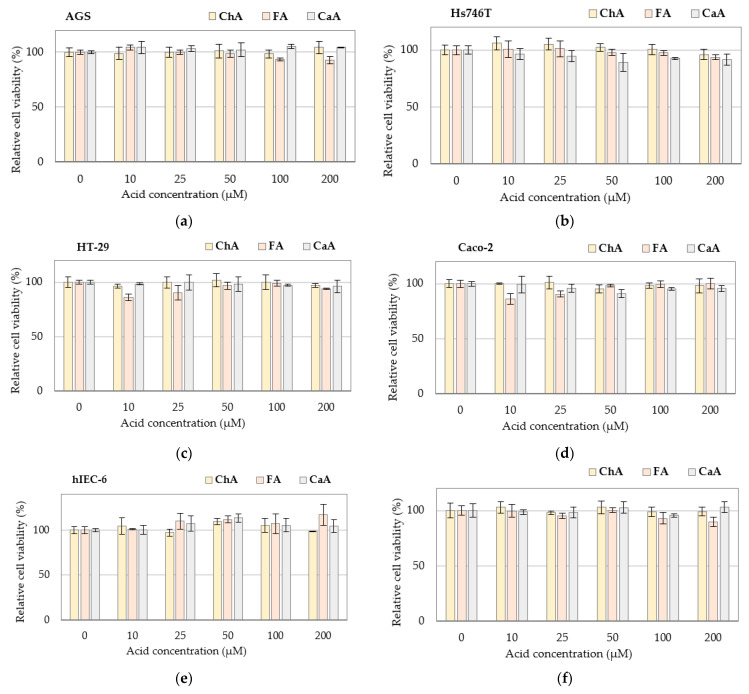
Cytotoxicity of phenolic acids against cancer and normal cells: (**a**) gastric cancer AGS cells; (**b**) gastric cancer Hs746T cells; (**c**) colon cancer HT-29 cells; (**d**) colon cancer Caco-2 cells; (**e**) normal small intestine hIEC-6 cells; (**f**) normal colon mucosa CCD 481 CoN cells determined by the MTT test. Changes in cell viability under acid treatment were expressed relative to control cell culture non-treated with phenolic acid tested. Values represent the means ± SD (n = 3). No statistical significance was detected. CaA–caffeic acid; ChA–chlorogenic acid; FA–ferulic acid.

Unlike GAs, PAs at concentrations up to 200 µM showed no cytotoxicity against the normal and cancer cells tested (Figure 2). This applies not only to CaA and FA but also to ChA, which, according to the literature data, inhibits the proliferation of A549 human cancer cells *in vitro* [30].

### 3.2. Changes in Bioaccessibility of Bioactive PJ Constituents during Gastrointestinal Digestion

We studied eight different materials, wherein four were fresh PJ samples, and the following four samples were the result of their processing. APJ, QAPJ, and VPJ were fresh PJ samples from three edible potato varieties prepared in the laboratory. IPJ was a fresh PJ obtained on an industrial scale as a side stream from the starch production line. The DPJ was a deproteinized fraction obtained from the production of feed potato protein (FPP) production line. MPP was a potato protein sample prepared in the laboratory, employing a membrane process [6]. NPP is a commercial product designed for human nutrition. The content of the key components of these preparations is presented in Table 2.

Edible varieties of potatoes differ from industrial varieties used for starch production, with a significantly lower content of GAs. It is the result of many years of breeders’ efforts to improve the quality of cultivated potatoes [42]. The variation in the content of bioactive compounds in different potato varieties has been repeatedly reported in the scientific literature. With regard to GAs, this differentiation is particularly high and may reach as much as five orders of magnitude [20]. In terms of meeting the requirements of the food law regarding the content of toxic GAs, only the juices of edible varieties (APJ, QAPJ, and VPJ) showed an acceptable level below 150 mg/kg. In that respect, the Agata variety was especially beneficial; APJ contained only a few micrograms of GAs per gram of dry mass. Among the products of PJ processing, only protein preparation (NPP) designed for human nutrition meets these requirements. In contrast, the highest GAs content was found in the supernatant after potato protein coagulation (DPJ). This product is currently recognized as waste and is subjected to biodegradation processes to reduce chemical oxygen demand and nutrient load in sewage [7,43]. The increased content of GAs in DPJ is related to the fact that the acidic environment reduces their affinity to proteins [44]. Nevertheless, FPP still contained much of the GAs. In this case, the protein preparation obtained in the membrane technology (MPP) turned out to be better, although fresh IPJ was used as a raw material for its production. This shows the potential of this technology to remove GAs from PJ. If the raw material used was PJ from a potato variety with low GAs content, such as Agata, it is possible to obtain a product that is safe from a toxicological point of view. The content of PAs in the tested samples was within the limits of the values reported so far in scientific studies.

Simulated gastric digestion processes caused only slight changes in the accessibility of the GAs from the products studied, which were mainly statistically insignificant (Table 2). Further gastrointestinal digestion had a more pronounced effect. Changes in GAs and PAs content observed in gastric and gastrointestinal digested products could be related mainly to pH values. However, enzymes and other digestive factors could also affect their bioaccessibility. Nevertheless, the Pearson correlation coefficient for gastric and gastrointestinal digestion indicates a linear relationship between the initial and final content of GAs (Table 3). The gastric digestion processes proved to have no effect on solanine and chaconine, as indicated by linear equation constants close to unity. Further digestion in the simulated gastrointestinal tract reduced the content of both GAs by approximately half.

### 3.3. Cytotoxicity of Gastrointestinally-Digested Potato Juice and Products of Its Processing

The PJ preparations were subjected to gastric and gastrointestinal digestion in the artificial gastrointestinal tract to obtain physiologically relevant data on their cytotoxicity to normal and cancer cells derived from the digestive system. The cytotoxic effects of gastric-digested PJ products were analyzed using stomach cancer AGS and Hs746T cell lines. In contrast, the cytotoxic potential of gastrointestinally-digested PJ products was evaluated using colon cancer HT-29 and Caco-2 cells, normal small intestinal hIEC-6 cells, and normal colon mucosa CCD 481 CoN cells.

Table 4 shows the IC_50_ doses that reduced cell viability and metabolic activity by 50% in applied cell culture models. The most potent cytotoxic effects on both normal and cancer cells were observed under treatment with digested DPJ, an industrial processing product of IPJ, distinguished by the highest cytotoxicity among fresh juice products. It is also worth noting that DPJ cytotoxicity is significantly higher against colon cancer HT-29 cells than against colon CCD 481 CoN cells derived from normal mucosa.

Agata and ‘Queen Anne’ variety PJ had the similar potential to decrease the viability of Hs746T and HT-29 cancer cells and normal intestinal hIEC-6 and CCD 481 CoN cells. However, the cytotoxic effects of APJ and QAPJ on normal cells were significantly lower than on colon cancer HT-29 cells. APJ and QAPJ differed in their cytotoxic activity towards stomach cancer AGS and colon cancer Caco-2 cells. Feed potato protein obtained by acid thermal coagulation of PJ was the most toxic protein preparation. In contrast, the protein designed for human nutrition revealed the lowest cytotoxic potential for all cell lines tested. Data in Table 2 and Table 4 suggest a possible correlation between cytotoxic potency and GAs content. The obtained results indicate the differences in cell susceptibility to treatment with PJ preparations. Colon cancer HT-29 cells were the most sensitive to the treatment, and stomach cancer Hs746T cells were the most resistant. Higher treatment resistance of Hs746T cells is most likely due to the metastasis stage of the cancer cells. This cell line originates from a diffuse gastric tumor metastasizing to the left leg muscle. While different PJ preparations varied in their biological activity, it should be noted that cancer cell lines were, on average, more susceptible than normal cell lines to the same products.

### 3.4. Statistical Considerations on the Bioactivity Products Studied

**Figure 3:**
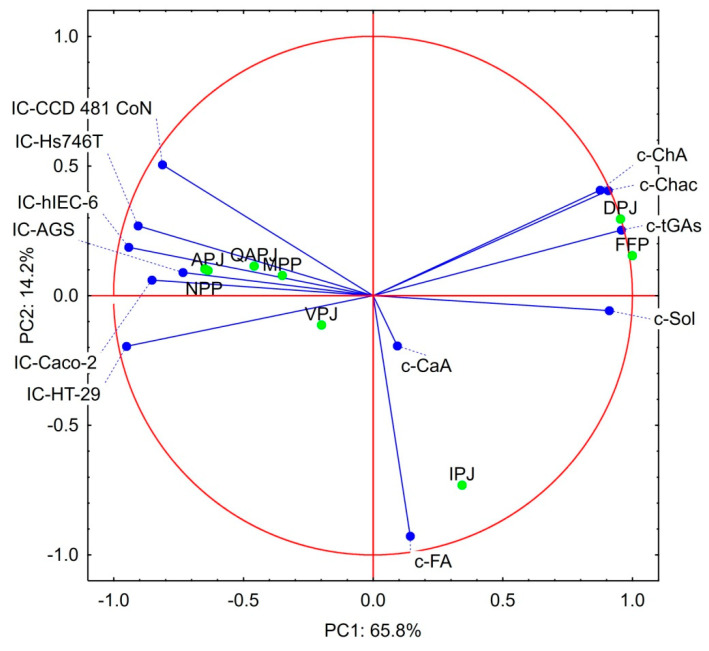
Principal component analysis of the data regarding the content of the bioactive substances in the raw materials and cytotoxicity of the products studied. Explanatory notes: IC-AGS; IC-Hs746T; IC-HT-29; IC-Caco-2; IC- hIEC-6; IC-CoN - IC_50_ values against AG IC-AGS; IC-Hs746T; IC-HT-29; IC-Caco-2; IC- hIEC-6; and CCD 481 CoN cell lines, respectively; c-CaA; c-ChA; c-FA; c-Sol; c-Chac; c-tGAs–contents of the caffeic acid; chlorogenic acid; ferulic acid; solanine; chaconine and total glycoalkaloids, respectively; APJ; QAPJ; VPJ; IPJ; DPJ; FPP; MPP; NPP—abbreviations describing potato juice products according to ‘Abbreviations’ section.

PCA analysis was performed to estimate which of the analytical data presented in Section 3.1, Section 3.2 and Section 3.3 are crucial for the bioactivity of the products studied (Figure 3). The results of this analysis can be considered legitimate as the two principal components (factors 1 and 2) explain 80.02% of the total variance. The cytotoxicity parameters of the materials studied (IC_50_ values) formed one group of lines located close to each other on the left side of the loading plot. The second group of lines located on the right side of the loading plot is formed by data describing the contents of solanine, chaconine, total GAs, and ChA. This observation pointed to the negative correlation between GAs contents and IC_50_ values, in other words indicating a positive correlation between cytotoxicity and GAs content, which was in line with expectations [23,24,25,26,27,28,29]. A strong positive correlation between ChA and chaconine was observed regarding the PAs content. It may be important for the biological activity of these substances in complex mixtures, such as fresh potato juices and their processing products. On the other hand, there was almost no correlation between FA and CA contents and other parameters studied. It indicates the key role of ChA in the bioactivity of plant materials mentioned in the literature [30,45,46,47,48]. Interesting conclusions could be drawn from the positions of the individual products on the biplot, as two groups could be distinguished. One group was formed by all juices acquired from edible potato varieties and by two protein preparations–NPP and MPP. The second group was formed by two products of industrial processing of PJ, i.e., FPP and DPJ. In contrast, IPJ differed from all others. Products’ scores on the biplot indicated that the resemblance of products belonging to the first group is due to the high IC_50_ values, while the similarity of the products from the second group is related to the high GAs content. IPJ is distinguished by a relatively high content of CA and FA. All these observations show that high cytotoxicity is not directly associated with the high content of GAs and that other factors also play a role.

An attempt to estimate the cytotoxicity of GAs in individual products studied was made, given the component scores of the individual products on the biplot, which suggest a more complex mechanism of cytotoxic activity of fresh PJ and products of its processing than simply the presence of GAs. For this purpose, the concept of effective IC_50_ values was created. This parameter considers the IC_50_ value presented in Table 4 and the content of the total GAs given in Table 2. The calculated data presented in Table 5 suggest completely different conclusions than those drawn on the basis of Table 4. Still, they are in line with the positions of individual products on the biplot (Figure 3). The GAs contained in PJ from the Agata variety turned out to be extremely effective against all analyzed cell lines. An exceptionally low content of GAs distinguished this variety. The increasing content of GAs in the juices of Queen Anne and Vivaldi varieties was paid for by the lower effectiveness of their activity on cells. This observation also concerned industrial juice, which is a mixture of several varieties with a higher content of starch and GAs than in edible potato varieties [6,7]. A similar relationship was observed in the potato protein preparations. The most effective were GAs in a protein preparation designed for human nutrition that meets the requirements of toxicological safety. Nevertheless, the earlier observation that the Hs746T cell line is the most resistant to the cytotoxic effects of the preparations studied has been confirmed. Special attention should be paid to DPJ as this preparation was distinguished by a favorable ratio of IC_50_ values against normal vs. cancer cell lines. A similar but less pronounced phenomenon in the case of FPP preparation could be observed. It is possible that this favorable ratio is caused by the presence of significantly larger amounts of ChA in these preparations compared to other materials studied. It is possible despite the fact that pure ChA did not show any cytotoxic activity in our analyses. The anticancer mechanism of ChA includes regulation of the expression of apoptosis-related factors and promotes apoptosis of cancer cells; influence on the cell division cycle and hindering of the reproduction, metastasis, and invasion of cancer cells; influence on the level of metabolism of cancer cells and their growth [45]. However, it should also be taken into consideration that PJ is a very complex material containing many different substances with potential bioactivity. Among them, vitamin C and group B vitamins should be mentioned first. The content of individual bioactive substances depends on the botanic variety and can vary even several levels of magnitude [7,20,49]. PJ processing causes further changes in the content of biologically active substances. Our experiment was limited to examining the effects of GAs and three PAs. This obviously limited the possibility of a precise assessment of the effect of individual bioactive substances. Nevertheless, the statistical analysis proved the synergistic effect of all bioactive substances present in the fresh juices and products of their processing. Moreover, higher effective cytotoxicity of total GAs in fresh juices than in processed preparations suggests important effects of vitamins and antioxidants, which are susceptible to degradation.

Behind the conception of an effective IC_50_ of total GAs in the samples studied (Table 5) was the attempt to try to compare these values with the IC_50_ of pure solanine and chaconine presented in Table 1 and expressed as μg/mL. If the effective IC_50_ values were found to be lower than the IC_50_ of the more toxic chaconine, this would indicate a synergistic effect of the bioactive components present in the analyzed samples. In fact, it turned out that this is the case with all juices from edible varieties. The food protein preparation NPP showed worse efficiency than pure chaconine, but only against the very resistant Hs746T line. However, special attention should be paid to DPJ. This preparation revealed an especially good ratio of cytotoxicity against cancer to normal cells. This is not only the truth in the case of the very resistant Hs746T cell line. This favorable ratio was also observed for the FPP. Based on our experiment, it is difficult to judge which features of both preparations determine their beneficial properties. Nevertheless, our results indicate the directions of research that should be undertaken in the future when looking for preparations that could be considered as part of anticancer therapy support.

**Figure 4:**
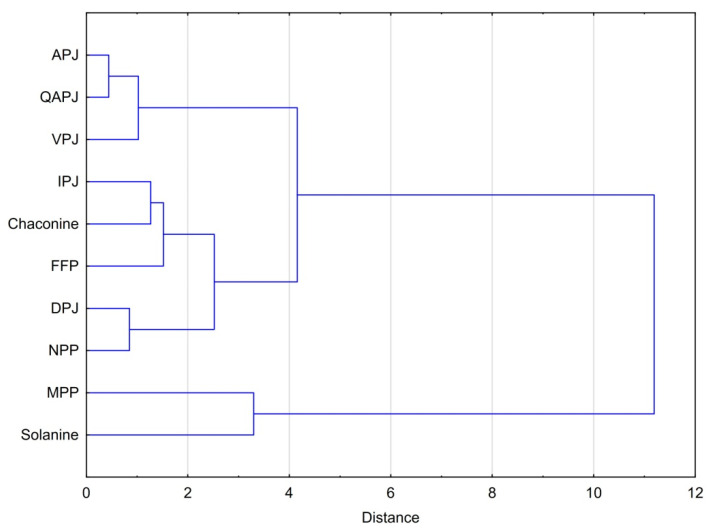
Dendrogram for hierarchical cluster analysis of cytotoxicity of potato juice and the products of its processing attributable to the total glycoalkaloids content. Explanation of the abbreviations used in the table can be found in the section ‘Abbreviations.’

The above-presented dependencies are even more visible in Figure 4. Considering the effective cytotoxicity of different materials against all cell lines studied, one can see that fresh juices obtained from the Agata and Queen Anne, followed by Vivaldi potato varieties, form a cluster of the most similar materials with the highest cytotoxicity per unit mass of total glycoalkaloids. The next cluster with slightly less effective cytotoxicity consists of NPP and DPJ. The aforementioned cluster is closely linked to the next one consisting of a by-product of starch production, feed protein, and pure chaconine. The last cluster consists of membrane protein and solanine with had highest effective IC_50_ values.

## 4. Limitations and Future Perspectives

The main goal of our work was to verify which bioactive components of PJ show antiproliferative activity. Eight different materials were studied. They include three samples of fresh juice derived from edible potato varieties; fresh PJ being a side stream from starch production line; a deproteinized fraction of PJ; feed potato protein obtained by acid-thermal coagulation, potato protein prepared by employing membrane technology and commercial potato protein designed for human nutrition. We examined the cytotoxicity of solanine and chaconine as well as the chlorogenic, ferulic, and caffeic acids. We also analyzed the bioavailability of the bioactive substances mentioned above after gastric and gastrointestinal digestion. Moreover, the cytotoxicity of PJ products subjected to gastric digestion was studied against AGS and Hs746T cell lines. Likewise, the cytotoxicity against the HT-29, Caco-2, hIEC-6, and CCD 481 CoN cell lines was analyzed with gastrointestinally-digested products. We have shown that the analyzed phenolic acids, including chlorogenic acid, do not show cytotoxicity to the cell lines used in the study. In contrast, both glycoalkaloids reveal significant cytotoxicity, which allowed for the calculation of IC_50_ against all cell lines. In all cases, chaconine was characterized by higher cytotoxicity than solanine.

Statistical analysis of the obtained results showed that fresh juices and, more generally, preparations with a low content of GAs are similar to each other in terms of IC_50_ values. In contrast, the similarity of other preparations is based on the content of solanine and chaconine. This suggested that the cytotoxicity of different materials is also determined by other factors, not only the content of the GAs itself. Therefore, the concept of the effective values of IC_50_ of GAs was created. This value describes the IC_50_ per unit mass of the total GAs contained in the sample. The calculation of the effective IC_50_ values for all analyzed samples and their comparison with the IC_50_ values of the pure GAs proved the synergistic effect of bioactive substances contained in PJ and the products of its processing. Our research does not make it possible to determine which substances are responsible for this synergism. One of the main questions is whether the ratio of solanine to chaconine is significant, and if so, what is its optimal value? Another important issue is which of the other bioactive substances influence the synergistic effect. Is it not only ChA but also other phenolic acids? What is the importance of vitamins? What is the molecular mechanism of these interactions? Could cellular and molecular pathways be mediated by reactive oxygen species (ROS) induced by DPJPs? The data reported in the literature indicates ROS involvement in antiproliferative PJ activity [50]. However, our study does not provide evidence to support this conception.

Interesting data obtained in this research are great inspiration for further detailed studies on molecular and cellular pathways via which fresh potato juice and products of its processing may interact with cancer cell proliferation and apoptosis. The promising findings also concern more potent inhibitory effects targeting cancer cells than normal colon mucosa cells. Unfortunately, more specific conclusions about the mechanisms of action of potato juice bioactive compounds cannot be drawn based on the scope of the experimental work presented and the analytical methods employed.

In future studies, it will be important to analyze the cell cycle progression and apoptosis or autophagy induction in the normal and neoplastic cells treated with physiologically relevant doses of potato juice bioactive constituents as single compounds, as combination mixtures, and as potato juice products. Future antiproliferative experiments should include cell cycle distribution by iodine propidine cell staining and flow cytometry measurement, as well as apoptosis analysis, including microscopic apoptosis detection preceding the analysis of caspase 3/7 activity, Annexin-V/PI staining assay, Tunnel assay, and pro-apoptotic and anti-apoptotic genes and proteins expression analysis using real-time PCR and Western Blotting methods.

Taking into account the variability of the composition of PJ related to biodiversity and seasonal changes, even the studies on fresh juices alone are a huge challenge for many research groups. Nevertheless, it would also be worth finding the reason for the favorable ratio of cytotoxicity of DPJ against cancer cells and against normal cells. Is it a result of changes in the content of individual bioactive substances, or is it an artifact of the deproteinization process itself? The industrial deproteinization process may result in the introduction of additional substances, sometimes undesirable from a nutritional point of view, which may act as some kind of adjuvant enhancing the potential cytotoxic effects of DPJ.

## 5. Conclusions

Our study proved that solanine and chaconine reveal cytotoxicity against cancer AGS, Hs746T, HT-29, Caco-2, and normal hIEC-6, CCD 481 CoN cell lines. In all cell cultures tested, chaconine was characterized by higher cytotoxicity than solanine, which was expressed by approximately twice lower IC_50_ values. In contrast, ferulic, caffeic, and chlorogenic acids at concentrations up to 200 µM do not show cytotoxicity to the cell lines used in the study.

Furthermore, fresh PJ and the products of its processing, subjected to gastric digestion, were shown to reveal cytotoxicity against stomach cancer AGS and Hs746T cells. Gastrointestinally-digested products revealed cytotoxicity against intestinal cancer HT-29 and Caco-2 cells and normal CCD 481 CoN and hIEC-6 cells. That cytotoxicity was non-linearly related to the presence of GAs in the analyzed products. The lowest cytotoxicity was found for juices derived from three edible potato varieties, the highest deproteinized potato juice, and the side stream of feed protein preparation production. However, statistical analysis showed that when considering the effectiveness of the mass unit of total GAs, the most effective are juices from edible varieties of potatoes containing less GAs and DPJ rich in GAs. Moreover, the cytotoxicity of a mass unit of total GAs contained in PJ and the products of its processing is higher than the cytotoxicity of chaconine. This proves synergy in the cytotoxic activity of biologically active compounds contained in PJ products.

## Figures and Tables

**Table 1:** Half maximal inhibitory concentration (IC_50_) of glycoalkaloids.

Cell Line	Solanine (μg/mL)	Chaconine (μg/mL)
AGS	10.26 ± 0.12 ^bc^	4.87 ± 0.29 ^c^
Hs746T	10.36 ± 0.49 ^c^	4.06 ± 0.03 ^abc^
HT-29	9.37 ± 0.13 ^b^	3.04 ± 0.85 ^ab^
Caco-2	9.64 ± 0.28 ^bc^	4.28 ± 0.06 ^bc^
hIEC-6	6.44 ± 0.28 ^a^	2.92 ± 0.55 ^a^
CCD 481 CoN	9.90 ± 0.46 ^bc^	3.67 ± 0.45 ^abc^

Values in columns marked with the same letter do not differ significantly (*p* > 0.05).

**Table 2:** Content of crucial bioactive components in the product studied.

Product	Glycoalkaloids (μg/g)			Phenolic Acids (μg/g)		
	Solanine	Chaconine	Total GAs	CaA	FA	ChA
APJ	3.11 ± 0.26 ^aA^	0.33 ± 0.07 ^aA^	3.44 ± 0.33	n.d.	1.92 ± 0.23 ^aC^	0.48 ± 0.06 ^aA^
APJ-G	3.26 ± 0.61 ^A^	1.18 ± 0.61 ^A^	4.44 ± 1.23	n.d.	0.07 ± 0.05 ^A^	0.56 ± 0.04 ^A^
APJ-GI	3.35 ± 0.62 ^A^	0.57 ± 0.05 ^A^	3.92 ± 0.68	n.d.	0.60 ± 0.01 ^B^	0.62 ± 0.12 ^A^
QAPJ	34.0 ± 2.8 ^aA^	25.3 ± 0.8 ^aA^	59.3 ± 3.7	n.d.	0.83 ± 0.01 ^aB^	0.46 ± 0.04 ^aA^
QAPJ-G	26.3 ± 0.2 ^A^	18.2 ± 4.2 ^A^	44.5 ± 4.3	n.d.	0.78 ± 0.01 ^A^	0.58 ± 0.02 ^B^
QAPJ-GI	25.5 ± 10.5 ^A^	17.4 ± 9.4 ^A^	42.9 ± 20.0	n.d.	0.90 ± 0.00 ^C^	0.68 ± 0.01 ^B^
VPJ	105 ± 6 ^aB^	146 ± 5 ^aC^	251 ± 11	0.80 ± 0.02 ^cA^	0.58 ± 0.01 ^aB^	0.23 ± 0.01 ^aA^
VPJ-G	109 ± 3 ^B^	124 ± 4 ^B^	233 ± 7	0.70 ± 0.10 ^A^	0.54 ± 0.01 ^A^	0.50 ± 0.12 ^B^
VPJ-GI	71.8 ± 1.9 ^A^	80.6 ± 2.4 ^A^	152 ± 4	n.d.	0.78 ± 0.00 ^C^	0.64 ± 0.01 ^B^
IPJ	675 ± 21 ^bcB^	101 ± 13 ^aA^	776 ± 28	0.47 ± 0.12 ^bA^	10.9 ± 1.7 ^bB^	0.49 ± 0.07 ^aA^
IPJ-G	770 ± 83 ^B^	180 ± 25 ^B^	859 ± 102	4.80 ± 5.80 ^A^	13.1 ± 0.3 ^B^	0.36 ± 0.07 ^A^
IPJ-GI	351 ± 4 ^A^	78.4 ± 7.8 ^A^	429 ± 12	n.d.	3.44 ± 0.12 ^A^	0.51 ± 0.04 ^A^
DPJ	984 ± 131 ^dB^	1621 ± 269 ^bB^	2605 ± 400	0.15 ± 0.01 ^aA^	0.51 ± 0.05 ^aB^	2.70 ± 0.14 ^bB^
DPJ-G	1001 ± 165 ^B^	1739 ± 156 ^B^	2740 ± 320	0.61 ± 0.06 ^B^	0.28 ± 0.01 ^A^	6.13 ± 0.04 ^C^
DPJ-GI	353 ± 43 ^A^	557 ± 12 ^A^	910 ± 55	n.d.	0.70 ± 0.03 ^C^	0.40 ± 0.01 ^A^
FPP	753 ± 178 ^cA^	1477 ± 469 ^bA^	2230 ± 646	0.72 ± 0.01 ^cA^	0.18 ± 0.00 ^aB^	3.50 ± 0.50 ^cB^
FPP-G	699 ± 38 ^A^	1479 ± 12 ^A^	2178 ± 49	0.65 ± 0.01 ^A^	0.08 ± 0.04 ^A^	5.12 ± 0.11 ^C^
FPP-GI	528 ± 37 ^A^	1051 ± 67 ^A^	1579 ± 104	n.d.	0.12 ± 0.02 ^A^	0.72 ± 0.01 ^A^
MPP	500 ± 43 ^bA^	3.63 ± 1.00 ^aA^	504 ± 44	0.75 ± 0.01 ^cA^	0.26 ± 0.01 ^aA^	0.42 ± 0.03 ^aB^
MPP-G	508 ± 16 ^A^	4.35 ± 2.90 ^A^	602 ± 19	0.77 ± 0.01 ^A^	0.22 ± 0.05 ^A^	0.19 ± 0.03 A
MPP-GI	495 ± 248 ^A^	6.30 ± 3.10 ^A^	501 ± 251	n.d.	0.64 ± 0.01 ^B^	0.68 ± 0.01 ^C^
NPP	43.1 ± 1.0 ^aA^	23.2 ± 4.8 ^aA^	66.3 ± 5.9	0.69 ± 0.06 ^cA^	0.14 ± 0.02 ^aA^	0.44 ± 0.02 ^aA^
NPP-G	156 ± 6 ^B^	19.9 ± 2.7 ^A^	176 ± 9	0.86 ± 0.09 ^A^	0.73 ± 0.04 ^C^	0.76 ± 0.02 ^B^
NPP-GI	37.5 ± 21.8 ^A^	21.1 ± 10.9 ^A^	58.6 ± 32.7	n.d.	0.65 ± 0.03 ^B^	0.76 ± 0.01 ^B^

n.d.–not detected. Explanation of the abbreviations used in the table can be found in the section ‘Abbreviations’—product subjected to gastric (-G) and gastrointestinal (-GI) digestion. Values in columns marked with the same letter do not differ significantly (*p* > 0.05); between products (lower-case) and within treatment (upper-case).

**Table 3:** Pearson correlations between the GAs content in raw materials and after digestion.

Correlation	r	*p*	Equation
Solanine content in the raw material (S) vs. solanine content after gastric digestion (S_G_)	0.98988	<0.0001	S_G_ = 0.98232 × S + 28.765
(S) vs. solanine content after gastrointestinal digestion (S_GI_)	0.85662	0.0066	S_GI_ = 0.48459 × S + 48.034
Chaconine content in the raw material (Ch) vs. chaconine content after gastric digestion (Ch_G_)	0.99831	<0.0001	Ch_G_ = 1.0375 × Ch + 5.1128
(Ch) vs. chaconine content after gastrointestinal digestion (Ch_GI_)	0.91727	0.0013	Ch_GI_ = 0.50155 × Ch + 13.958

**Table 4:** Cytotoxicity of potato juice and products of its processing subjected to the gastric (-G) and gastrointestinal (-GI) digestion expressed as IC_50_ concentration (mg/mL).

Cell Line	PJ and Products of its Processing Subjected to the Gastric (-G) Digestion							
	APJ-G	QAPJ-G	VPJ-G	IPJ-G	DPJ-G	FPP-G	MPP-G	NPP-G
AGS	14.39 ± 0.51 ^a^	9.20 ± 0.22 ^c^	11.40 ± 0.05 ^e^	1.90 ± 0.13 ^f^	0.061 ± 0.001 ^h^	1.00 ± 0.12 ^g^	11.40 ± 0.05 ^b^	2.74 ± 0.13 ^d^
Hs746T	30.49 ± 0.03^b^	30.68 ± 2.57 ^b^	11.45 ± 0.09 ^d^	4.86 ± 0.07 ^e^	2.57 ± 0.07 ^e^	3.01 ± 0.02 ^e^	20.73 ± 0.84 ^c^	36.49 ± 1.42 ^a^
**Cell Line**	**PJ and Products of its Processing Subjected to the Gastrointestinal (-GI) Digestion**							
	**APJ-GI**	**QAPJ-GI**	**VPJ-GI**	**IPJ-GI**	**DPJ-GI**	**FPP-GI**	**MPP-GI**	**NPP-GI**
HT-29	7.16 ± 0.03 ^b^	6.60 ± 0.22 ^b^	5.97 ± 0.33 ^b^	4.90 ± 0.12 ^c^	0.087 ± 0.02 ^e^	0.25 ± 0.03 ^d^	5.11 ± 0.20 ^c^	9.19 ± 1.56 ^a^
Caco-2	11.34 ± 0.33 ^b^	5.24 ± 0.14 ^d^	8.22 ± 0.87 ^c^	4.70 ± 0.13 ^d^	1.75 ± 0.16 ^e^	1.84 ± 0.12 ^e^	12.23 ± 0.15 ^b^	14.23 ± 0.25 ^a^
hIEC-6	12.92 ± 0.06 ^b^	14.95 ± 0.13 ^b^	7.61 ± 0.08 ^c^	5.94 ± 0.08 ^c^	2.19 ± 0.14 ^d^	3.04 ± 0.15 ^d^	15.04 ± 0.10 ^b^	16.07 ± 0.96 ^a^
CCD 481 CoN	11.34 ± 0.31 ^b^	10.58 ± 0.58 ^b^	7.93 ± 0.53 ^c^	7.57 ± 0.05 ^c^	3.06 ± 0.09 ^d^	2.23 ± 0.06 ^d^	10.92 ± 0.21 ^b^	14.72 ± 0.96 ^a^

An explanation of the abbreviations is shown in the section ‘Abbreviations.’ Values marked with the same letter do not differ significantly within the cell line (*p* > 0.05).

**Table 5:** The cytotoxicity of potato juice and the products of its processing attributable to total glycoalkaloids content.

Cell Line	PJ and Products of Its Processing Subjected to the Gastric (-G) Digestion							
	APJ-G	QAPJ-G	VPJ-G	IPJ-G	DPJ-G	FPP-G	MPP-G	NPP-G
AGS	0.06	0.41	1.35	1.63	0.17	2.17	6.86	0.48
Hs746T	0.14	1.37	2.67	4.17	7.03	6.56	12.48	6.42
**Cell Line**	**PJ and Products of Its Processing Subjected to the Gastrointestinal (-GI) Digestion**							
	**APJ-GI**	**QAPJ-GI**	**VPJ-GI**	**IPJ-GI**	**DPJ-GI**	**FPP-GI**	**MPP-GI**	**NPP-GI**
HT-29	0.03	0.28	0.91	2.10	0.08	0.40	2.56	0.54
Caco-2	0.04	0.22	1.25	2.02	1.59	2.91	5.88	0.83
hIEC-6	0.05	0.64	1.16	2.55	1.99	4.0	7.541	0.94
CCD 481 CoN	0.04	0.45	1.21	3.25	2.78	3.52	5.47	0.86

Explanation of the abbreviations used in the table can be found in the section ‘Abbreviations.’

## Data Availability

The raw data required to reproduce these findings cannot be shared at this time as the data also forms part of an ongoing study.

## Abbreviations

APJ	Fresh juices from potatoes of ‘Agata’ variety
APJ-G	APJ after digestion in the stomach
APJ-GI	APJ after complete gastrointestinal digestion
CaA	Caffeic acid
ChA	Chlorogenic acid
DPJ	Deproteinized potato juice
DPJ-G	DPJ after digestion in the stomach
DPJ-GI	DPJ after complete gastrointestinal digestion
FA	Ferulic acid
FPP	Feed potato protein from PPZ Trzemeszno S.A. (Trzemeszno, Poland)
FPP-G	FPP after digestion in the stomach
FPP-GI	FPP after complete gastrointestinal digestion
GAs	Potato glycoalkaloids
IC_50_	The half-maximal inhibitory concentration
IC-AGS	IC_50_ against AGS cell line
IC-Hs746T	IC_50_ against Hs746T cell line
IC-HT-29	IC_50_ against HT-29 cell line
IC-Caco-2	IC_50_ against Caco-2 cell line
IC- hIEC-6	IC_50_ against hIEC-6 cell line
IC-CCD 481 CoN	IC_50_ against CCD 481 CoN cell line
IPJ	Industrial potato juice
IPJ-G	IPJ after digestion in the stomach
IPJ-GI	FPP after complete gastrointestinal digestion
MPP	Potato juice protein concentrate obtained by membrane processing of potato juice
MPP-G	MPP after digestion in the stomach
MPP-GI	FPP after complete gastrointestinal digestion
NPP	Solanic^®^200 from Avebe (Veendam, The Netherlands)
NPP-G	NPP after digestion in the stomach
NPP-GI	FPP after complete gastrointestinal digestion
PAs	Phenolic acids
PCA	Principal component analysis
PDPJPs	Post-digestion potato juice and products of its processing
PJ	Potato juice
QAPJ	Fresh juices from potatoes of the ‘Queen Anne’ variety
QAPJ-G	QAPJ after digestion in the stomach
QAPJ-GI	FPP after complete gastrointestinal digestion
VPJ	Fresh juices from potatoes of the ‘Vivaldi’ variety
VPJ-G	VPJ after digestion in the stomach
VPJ-GI	FPP after complete gastrointestinal digestion
